# Insecticide resistance levels, spatial distribution, and *kdr* mutations in the dengue vector *Aedes albopictus* of Hong Kong

**DOI:** 10.1371/journal.pntd.0013792

**Published:** 2025-12-22

**Authors:** Shaolin Han, Elliott F. Miot, Yunshi Liao, Munsif Ali Khan, Mathilde Rivot, Lilia Tsz-Wing Tang, Jehan Zeb, Ka Mei Szeto, Long Ching, Tsz Him Li, Xintong Huang, Brinna E. L. Barlow, Sebastien Marcombe, Tommy Tsan-Yuk Lam

**Affiliations:** 1 Centre for Immunology & Infection, Hong Kong SAR, China; 2 State Key Laboratory of Emerging Infectious Diseases, School of Public Health, The University of Hong Kong, Hong Kong SAR, China; 3 MIVEGEC, Université de Montpellier, IRD, CNRS, Montpellier, France; 4 Vector Control Consulting—South East Asia Sole Co., Ltd., Vientiane, Laos; 5 Laboratory of Data Discovery for Health, Hong Kong SAR, China; 6 HKU-Pasteur Research Pole, Hong Kong SAR, China; 7 The Hong Kong Jockey Club Global Health Institute, The University of Hong Kong, Hong Kong SAR, China; CNRS: Centre National de la Recherche Scientifique, FRANCE

## Abstract

*Aedes albopictus* (Skuse), a primary dengue vector in Hong Kong, poses significant challenges to public health due to escalating insecticide resistance in Asia. To address this concern, we evaluated resistance profiles of five field-derived *Ae. albopictus* populations across Hong Kong using WHO insecticide susceptibility bioassays. Metabolic detoxification using synergists test with piperonyl butoxide (PBO) and target-site mutations referred to as knockdown resistance (*kdr*) in the voltage-gated sodium channel (VGSC) gene were characterized to elucidate resistance mechanisms. The results show that *Ae albopictus* populations in Hong Kong exhibited high resistance to commonly used pyrethroids (permethrin, deltamethrin) and the organochlorine dichlorodiphenyltrichloroethane (DDT), as well as emerging resistance to the organophosphate malathion. Resistance to three larvicides, temephos, spinosad, and pyriproxyfen, is likely to be developing, whereas *Bacillus thuringiensis israelensis* (*Bti*) still retains efficacy. Sequencing revealed the F1534S/L mutation in VGSC Domain III (29.0% mutation frequency), strongly correlated with pyrethroid survivorship. A minor dual mutation (S406T + L424H) in Domain I was recorded but showing limited phenotypic association. Synergist assays demonstrated partial or full restoration of pyrethroid susceptibility with PBO, implicating cytochrome P450-mediated metabolic resistance as a co-factor. These findings confirm that *Ae. albopictus* in Hong Kong has evolved multifaceted resistance mechanisms, driven by both target-site mutations and metabolic detoxification. This study provides critical evidence to optimize local vector control strategies and contributes resistance profiles to the Asia-Pacific region, aiding regional efforts to mitigate dengue transmission risks.

## Author summary

*Aedes albopictus* is a major vector for dengue in Hong Kong, and rising insecticide resistance threatens the effectiveness of current control measures. This study evaluated resistance in five mosquito populations across the territory, revealing resistance to commonly used pyrethroids and dichlorodiphenyltrichloroethane (DDT) and emerging resistance to malathion. There is evidence of emerging resistance to temephos, spinosad, and pyriproxyfen, whereas *Bacillus thuringiensis israelensis* (*Bti*) efficacy persists. Genetic analysis identified the F1534S/L mutation in the voltage-gated sodium channel (VGSC) gene as strongly associated with pyrethroid resistance, alongside evidence of metabolic resistance mediated by cytochrome P450 enzymes. These findings align with trends in neighboring regions, highlighting a broader resistance hotspot in southern China and Southeast Asia. The results underscore the urgent need for sustained resistance monitoring and the adoption of more robust vector management strategies, such as rotating insecticides with different modes of action, integrating synergists like piperonyl butoxide (PBO), emphasizing biological control, and adopting emerging alternative insecticides approved by WHO. This research provides essential data to guide local and regional efforts to mitigate dengue transmission risks posed by resistant mosquito populations.

## Introduction

The *Aedes albopictus* (Skuse) mosquito, commonly known as the Asian tiger mosquito, is a globally invasive species and a vector of significant public health concern [1–3]. Native to Southeast Asia, this species has demonstrated a memorable ability to adapt to diverse environments, facilitating its spread across tropical, subtropical, and even temperate regions [4,5] including Hong Kong [6,7]. *Aedes albopictus* is notorious for its aggressive daytime biting behavior and serves as a primary vector for several arboviruses, including dengue fever, chikungunya, and Zika virus [8–10]. Clinical manifestations range from febrile illness to severe dengue with hemorrhage fever and shock [11]; chikungunya can cause acute, and sometimes chronic, polyarthralgia and myalgia [12]; and Zika commonly presents with fever, rash, joint and muscle pain, red eyes, and headaches [13]. Moreover, as an opportunistic species, *Ae. albopictus* acts as an efficient bridge vector for at least 14 zoonotic arboviruses, in addition to the above three [14]. This adaptability and broad vector competence pose an escalating threat to public health systems worldwide [15–18].

*Aedes albopictus* was first documented in Hong Kong in 1926 by Severn [19], and it has since become the most common in this region, constituting a major public health risk due to its role as a main vector of numerous diseases. Over the past decades, Hong Kong has experienced periodic outbreaks of dengue [20–23]. The first major recorded outbreak occurred in 2002, involving 19 locally transmitted cases [20]. Another significant outbreak took place in 2018, when 29 local cases were reported, marking the largest local outbreak in recent decades [20]. In 2024, 156 imported and 5 local cases have been reported [23]. These outbreaks underscore the ongoing threat posed by this species in the region. Additionally, Hong Kong’s geographical and economic characteristics increase its vulnerability to transmissible mosquito-borne diseases. For example, situated on China’s southern coast and connected to Guangdong Province, where epidemic dengue and other mosquito-borne diseases, such as chikungunya, are recurrent [24–26], Hong Kong faces heightened risks. As a trade and travel hub, the risk of mosquito-borne pathogens being introduced from neighboring Southeast Asian countries and causing local transmission also are prominent [27,28].

The widespread use of insecticides has led to a growing resistance in *Aedes* mosquitoes worldwide [29,30], significantly undermining the effectiveness of vector control strategies [16,18,31]. Resistance of *Aedes* mosquitoes to dichlorodiphenyltrichloroethane (DDT), the first commercially used insecticide, has been a global issue, despite its ban in many countries since the last century [32,33]. Following the development of organophosphates, resistance spread across the Americas, Southeast Asia, and Africa [32]. Subsequently, as pyrethroids became extensively used due to their lower environmental impact and reduced risk to users, resistance to pyrethroids has proliferated even more rapidly worldwide [32]. While insect growth regulators (IGRs) and microbial insecticides have generally remained effective, emerging concerns have been noted. A recent study reported that *Ae. albopictus* mosquitoes in China have developed resistance to pyriproxyfen [34]. Additionally, a tolerance to IGRs was observed in *Aedes* mosquitoes in both Malaysia and the United States [35,36]. In Hong Kong, the widespread adoption of synthetic insecticides for mosquito control began with the introduction of DDT, which was banned in 1987 [37,38]. Contemporary mosquito control programs utilize a diverse array of insecticides, including pyrethroids, organophosphates, carbamates, *Bacillus thuringiensis israelensis* (*Bti*), spinosad, and IGRs (e.g., pyriproxyfen) [39]. Despite the global urgency of monitoring insecticide resistance, data on resistance levels in *Ae. albopictus* mosquitoes in Hong Kong remain strikingly limited, underscoring a critical gap in local vector management strategies.

Several mechanisms have driven insecticide resistance in mosquitoes. One of the primary mechanisms is target site mutations in the voltage-gated sodium channel (VGSC) gene, referred to as knockdown resistance (*kdr*) [40]. Mutations in this gene can reduce the sensitivity of mosquitoes to pyrethroids and DDT, which functions as a sodium channel modulator [41]. The first *kdr* mutation, F1534C, in *Ae. albopictus* was detected in Singapore [42]. Since then, mutations I1532T, F1534 (C, S, or L) and V1016G, have been sequentially reported in this species in various countries in Asia, Europe, and the Americas, including Brazil, Bulgaria, China, France, Georgia, Italy, Malta, Romania, Spain, Switzerland, the United States, Turkey, and Vietnam [43,44]. Multiple mutations have been reposted on VGSC that enhance resistance, such as the S989P + V1016G + F1534C triple mutations [45] and the concomitant mutations L982W + F1534C and V1016G + F1534C [46], which exhibited extremely high level of resistance to pyrethroid in *Ae. aegypti* mosquitoes. In addition to target site mutations, enhanced detoxification enzymes also contribute to insecticide resistance [16,18,47]. Overexpression or increased activity of detoxification enzymes allows mosquitoes to metabolize and neutralize insecticides more effectively [48]. Cytochrome P450 monooxygenases (P450s) are critical for detoxification of pyrethroids in *Aedes*, *Anopheles*, and *Culex* mosquitoes [49–51]. Notably, the presence of piperonyl butoxide (PBO), an oxidase inhibitor, can inhibit many P450 enzymes, thus restoring the effectiveness of pyrethroids [52,53]. The use of PBO has practical implications in pest management and diagnosis to determine P450s involvement [54–56]. Understanding these mechanisms is crucial for developing effective mosquito control strategies and mitigating the associated public health risks.

**Fig 1:**
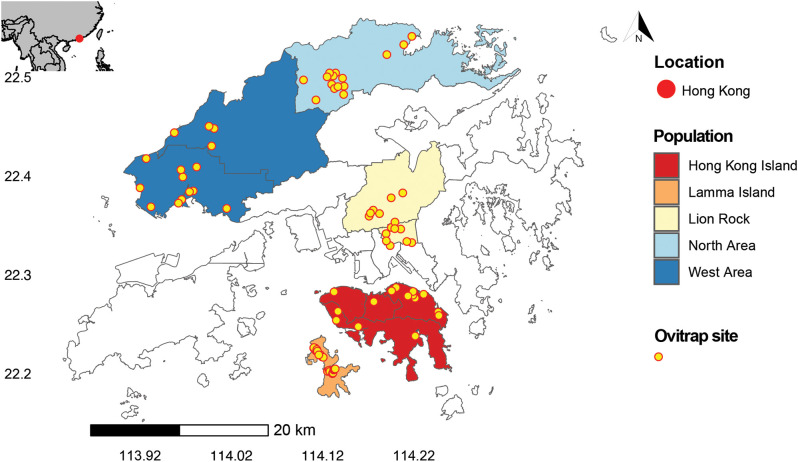
Map of Hong Kong showing sampling locations and ovitrap distribution. Colored areas indicate the field populations included in the study; yellow dots mark ovitrap sites. The base map layer was derived from DATA.GOV.HK and reprocessed by Esri China (HK) Ltd. (used with permission; https://opendata.esrichina.hk/datasets/hong-kong-18-districts/explore). The map was generated on R (version 4.3.1) [57] using the sf package [58].

This study aims to address key gaps in our understanding of insecticide resistance in different *Ae. albopictus* populations in Hong Kong. The primary objective was to determine the insecticide resistance status of *Ae. albopictus* across the full latitudinal range of the species in the region. Specifically, we examined populations from five areas, including two islands and three inland populations (Fig 1). We selected eight insecticides that represent the main classes of insecticides historically or currently used for mosquito control in Hong Kong (Table 1). Additionally, we investigated the function of PBO in restoring resistance to pyrethroids and examined the key *kdr* mutations on the VGSC gene involved in this resistance. By providing a comprehensive assessment of insecticide resistance in *Ae. albopictus* populations, this study informed future mosquito control strategies and policies, ultimately contributing to improved public health outcomes in Hong Kong and neighboring regions.

**Table 1:** Type, class, and mechanism of all insecticides tested in this study.

Type	Insecticide	Class	Mechanism
Adulticide	Deltamethrin	Pyrethroid	Sodium channel modulator
	Permethrin		
	DDT	Organochlorine	
	Malathion	Organophosphate	Acetylcholinesterase inhibitor
Larvicide	*Bti*	Biolarvicide	Cell membrane destruction
	Spinosad	Naturalyte	Nicotinic acetylcholine receptor
	Temephos	Organophosphate	Acetylcholinesterase inhibitor
	Pyriproxyfen	Insect growth regulator	Juvenile hormone mimics

## Methods

### Mosquitoes and field collection

Mosquito populations were established from larvae collected in five areas across Hong Kong (Fig 1). They are Hong Kong Island, Lamma Island, Lion Rock, North Area, and West Area, with population density (persons/km^2^) of 14963, 340.9, 26861, 2276, and 5315.5, respectively (data from https://www.citypopulation.de/en/china/cities/hongkong/). In each area, 15 ovitraps (23 ovitraps on Lamma Island) were strategically placed (see specific locations in S1 Table), each consisting of a black bucket (1L) filled with pure water, minor soil, and leaf litter to provide essential nutrients for larvae development. Larvae were collected on a weekly basis, transported back to the insectarium, and sorted to the genus level. The *Aedes* larvae were then reared to adulthood using ground fish food (Tetra Rubin), and *Ae. albopictus* adults were selected and identified with a mouth aspirator with HEPA Filter (Model 612, John W. Hock) and then transported into new cages for mating. *Aedes albopictus* females were fed heparin sodium anticoagulant rabbit blood (Guangzhou Ruite Biotechnology Co., Ltd) using Hemotek membrane feeding apparatus for two hours, three times a week. The larvae collection process was maintained for approximately two months to ensure an adequate number of *Ae. albopictus* adults for the tests. The collection period spanned from 2022 to 2024, intermittently, to obtain a sufficient sample size across the study area and were not stratified by season. For each population, we reared the mosquitoes to the F2 or F3 generations to ensure a stable population for subsequent bioassays. A susceptible strain of *Ae. albopictus* was obtained from Centers for Disease Control and Prevention for distribution by BEI Resources, NIAID, NIH, USA: *Ae. albopictus*, Strain ATM-NJ95, Eggs, NR-48979. Mosquitoes were reared in incubators at 28°C and 75% RH, with a 14:10 light-dark photoperiod.

### Larval bioassays

Insecticides were prepared by diluting active ingredients obtained from Merck (Darmstadt, Germany) in ethanol to the desired concentrations, following WHO guidelines [59]. The insecticides evaluated included temephos (6.25 mg/L, absolute ethanol-denatured + 2% butanone, Vector Control Research Unit, Universiti Sains Malaysia, Malaysia), spinosad (99.6%, powder, Supelco, Germany), and pyriproxyfen (98.1%, powder, Sigma-Aldrich, Germany) (Table 1). *Bti* (Table 1), H14 (10.2% w/w), was also tested as an aqueous suspension concentrate (Joy Time International Ltd, Hong Kong), using water for dilution. All bioassays were conducted on late third- and early fourth-instar larvae of *Ae. albopictus*.

For each bioassay, 25 larvae from each population were transferred to plastic cups containing 99 mL of distilled water and 1 mL of insecticide at the specified concentration. Four cups per concentration (100 larvae in total) were prepared for each population, with an additional 100 larvae for the ATM-NJ95 susceptible reference strain. Insecticide concentrations were tested across 4–8 levels, spanning an activity range of 10% to 95% mortality. Each test was replicated at least three times. Control treatments consisted of 99 mL of distilled water and 1 mL of ethanol, or 100 mL of distilled water alone when testing with *Bti*. Larval mortality was recorded 24 hours post-exposure, except for pyriproxyfen, where mortality was assessed every 24 hours until emergence due to the delayed action of this insect growth regulator. In these cases, larvae were provided with food daily at 100 mg/L. All bioassays were conducted at a constant temperature of 28°C in an incubator with a 14:10 light-dark photoperiod.

### Adult bioassays

#### Susceptibility tests.

All bioassays on adult *Ae. albopictus* were conducted according to standard WHO guidelines [60]. Briefly, 25 3-to-6-day-old female mosquitoes were placed in WHO test tubes and allowed to acclimate for 1 hour. Four tubes per insecticide were used, totaling 100 mosquitoes for each population and an additional 100 for the ATM-NJ95 susceptible reference strain. Mosquitoes were exposed to insecticide-treated papers for 1 hour. These papers, impregnated with specific insecticide concentrations, were sourced from the Vector Control Research Unit at the University of Science, Penang, Malaysia. The following diagnostic doses were tested: 4% DDT (organochlorine), 5% malathion (organophosphate), 0.03% deltamethrin, and 0.75% permethrin [61–63] (both pyrethroids) (Table 1). After exposure, mosquitoes were transferred into holding tubes containing 10% sugar solution and kept in a climate chamber set at 28°C with 80% relative humidity and a 14:10 light-dark cycle. Mortality was assessed 24 hours post-exposure. Control groups were included for each insecticide family, with four tubes treated using control papers coated with risella oil for DDT, olive oil for malathion, and silicone oil for the others, following the same protocol. Each test was replicated three times. According to WHO criteria, a population is considered resistant if mortality after 24 hours is under 90%, suspected of resistance if mortality is between 90% and 98%, and susceptible if mortality is over 98%.

#### Synergist tests.

Additionally, we evaluated the potential of PBO to restore pyrethroid susceptibility in adult mosquitoes, following WHO guidelines [64]. Using the same protocol described above, mosquitoes underwent an additional 1-hour pre-exposure to papers impregnated with 4% PBO before exposure to either the pyrethroid insecticides or the associated silicone oil control. All mosquitoes were immediately stored at 80°C after the completion of the adult bioassays.

### DNA extraction

Genomic DNA was extracted individually from *Ae. albopictus* samples collected from adult bioassays (both dead and alive) with the two pyrethroids across the populations of Hong Kong Island and Lamma Island using *Quick*-DNA 96 Plus Kit (ZYMO Research, USA), following manufacturer’s DNA extraction protocols. The quality/quantity of extracted DNA was assessed using a Qubit Fluorometer (Thermo Fisher Scientific).

### *kdr* detection

#### PCR amplification.

Partial nucleotide sequences of transmembrane segment 6 of each VGSC domain (I-IVTm6) were amplified via polymerase chain reaction (PCR) using predesigned primer sets [65] (S2 Table). PCRs were performed using TaKaRa Taq DNA Polymerase Hot Start PCR kit (TaKaRa Bio Inc). Each reaction was carried out in a total volume of 30 µl containing 0.15 µl of TaKaRa Ex Taq HS, 3 µl of 10X Ex Taq Buffer, 2.4 µl of dNTP Mixture, 0.5 µl of each primer (forward and reverse), 2 µl Template DNA and 21.45 µl of ddH_2_0. Thermocycling conditions were optimized for VGSC target sites (S2 Table). The PCR products were confirmed by gel electrophoresis using 3% agarose gel and purified with AMPure XP reagent (Beckman Coulter Life Sciences)

#### Sequencing and analysis.

The amplified PCR products were sequenced with BGI Genomics (Hong Kong) using predesigned sequencing primers in both forward and reverse directions [65] (S2 Table). The resulting chromatogram files were edited using SeqMan software (DNASTAR, Madison, WI, USA). The query dataset was aligned with reference VGSC gene sequences from *Ae. albopictus* in GenBank using MAFFT [66] for variant detection and annotation across the amplified segments of the VGSC gene. All sequenced amplicons from VGSC I-IVTm6 were analyzed for both known and novel nonsynonymous mutations that could confer resistance to pyrethroids [43,65,67–72].

### Statistical analysis

Data from larvicide bioassays were analyzed by log-transforming concentrations. For pyriproxyfen, emergence inhibition (IE; proportion of adults prevented from emerging) was calculated. Outliers were removed for each population-insecticide combination using Cook’s distance. Probit regression models were fitted to estimate lethal concentrations (LC_50_ and LC_95,_ or IE_50_ and IE_95_). Resistance Ratios, RR_50_ and RR_95,_ were then calculated by dividing the LC_50_ (IE_50_) and LC_95_ (IE_95_) values of the tested population by those of the reference strain, respectively. Data from susceptibility tests were analyzed using linear mixed-effects models with insecticide type and human population density as fixed effects, their interaction, and mosquito population as a random effect, and significance was determined using Type III ANOVA for fixed effects and likelihood ratio tests for random effects. The effect of PBO was analyzed using linear mixed-effects models with PBO pre-exposure (yes/no) as a fixed effect and mosquito population as a random effect. Separate models were fitted for deltamethrin and permethrin exposures. Associations between the genotypes (mutant/wild) and phenotypes (live/dead) were analyzed using the Chi-square test of independence. We computed the odds ratio (OR) and its 95% confidence interval (CI) to quantify the association. All data were analyzed and visualized on R (version 4.3.1) [57] with the drc [73] and ggplot2 [74] packages.

## Results

### Larval bioassays

The results of the larvae bioassays and dose-response curves are summarized in Table 2 and S1 File, respectively. Resistance ratios (RR_50_) for all *Ae. albopictus* populations across Hong Kong to *Bti*, spinosad, temephos, and pyriproxyfen (IGR) range from 0.29–1.38, 0.86–3.42, 1.42–3.31, and 0.88–3.40, respectively. Analysis reveals initial susceptibility at the LD_50_ level (RR_50_ < 5) across all populations. However, resistance is evident at the LD_95_ level in both Hong Kong Island and Lion Rock populations. Both populations exhibit moderate resistance to temephos, with RR_95_ values of 5.21 and 6.35 (5 < RR < 10), respectively. Notably, high-level resistance was observed to pyriproxyfen in the Hong Kong population (RR_95_ = 12.5) and to spinosad in the Lion Rock population (RR_95 _= 12.08). Original data were provided in S3 Table.

**Table 2:** Lethal dose (expressed in mg/L) and resistance status of *Ae. albopictus* larvae against *Bti*, spinosad, temephos, and pyriproxyfen.

Larvicide		Population					
		ATMNJ 95	Hong Kong Island	Lamma Island	Lion Rock	West New Territories	North New Territories
*Bti*	LD50 (95% CI)	0.13 (±0.01)	0.17 (±0.02)	0.15 (±0.01)	0.18 (± 0.01)	3.81E-2 (±2.48E-3)	5.51E-2 (±3.28E-3)
	LD95 (95% CI)	0.32 (±0.03)	0.42 (±0.04)	0.34 (±0.03)	0.31 (±0.02)	0.11 (±6.97E-3)	0.15 (±8.86E-3)
	RR50	–	1.31	1.15	1.38	0.29	0.42
	RR95	–	1.31	1.06	0.97	0.34	0.46
Spinosad	LD50 (95% CI)	0.07 (±0.01)	0.09 (±0.01)	0.06 (±0.01)	0.24 (±0.03)	0.13 (±8.48E-3)	0.10 (±6.98E-3)
	LD95 (95% CI)	0.12 (±0.01)	0.14 (±0.01)	0.24 (±0.02)	1.45 (±0.17)	0.40 (±2.65E-2)	0.21 (±1.49E-2)
	RR50	–	1.29	0.86	3.42	1.86	1.43
	RR95	–	1.17	2	12.08	3.33	1.75
Temephos	LD50 (95% CI)	3.96E-3 (±3.60E-4)	1.31E-2 (±1.21E-3)	7.93E-3 (±5.40E-4)	5.62E-3 (±1.79E-3)	8.59E-3 (±7.40E-4)	1.11E-2 (±9.30E-4)
	LD95 (95% CI)	8.02E-3 (±7.30E-4)	4.18E-2 (±3.87E-3)	1.60E-2 (±1.09E-3)	5.09E-2 (±1.62E-2)	1.79E-2 (±1.54E-3)	2.12E-2 (±1.77E-3)
	RR50	–	3.31	2.00	1.42	2.17	2.80
	RR95	–	5.21	2.00	6.35	2.23	2.64
Pyriproxyfen	LD50 (95% CI)	4.50E-5 (±5.63E-5)	1.53E-4 (±2.31E-4)	4.40E-5 (±3.29-5)	7.86E-5 (±7.85E-5)	6.17E-5 (±4.55E-5)	3.95E-5 (±3.11E-5)
	LD95 (95% CI)	7.56E-4 (±9.48E-4)	9.45E-3 (±1.43E-2)	2.45E-4 (±1.83E-4)	7.33E-4 (±7.33E-4)	3.44E-4 (±2.53E-4)	1.79E-4 (±1.41E-4)
	RR50	–	3.40	0.98	1.75	1.37	0.88
	RR95	–	12.5	0.32	0.97	0.46	0.23

LD₅₀ and LD₉₅, 50% and 95% lethal dose, respectively; CI, confidence interval; RR₅₀ and RR₉₅, resistance ratio (calculated as LD of field population/ LD of ATMNJ95). Resistance classification: RR < 5 (Susceptible), 5–10 (Moderate), > 10 (High). *Bti*, *Bacillus thuringiensis israelensis*.

### Adult bioassays

**Fig 2:**
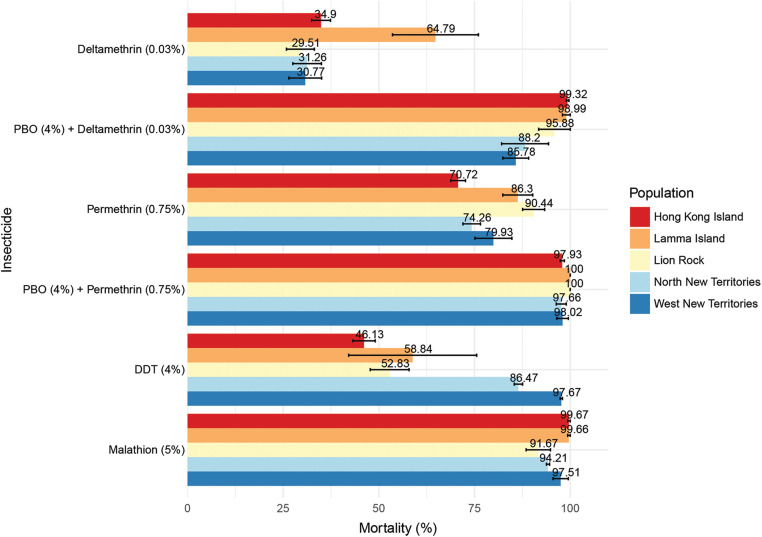
Mortality (%) of females of *Ae. albopictus* from Hong Kong after 24-hour exposure to selected insecticides). Females of *Ae. albopictus* were 1-hour exposured to deltamethrin, deltamethrin with 1-hour pre-exposure to piperonyl butoxide (PBO), permethrin, permethrin with 1-hour pre-exposure to PBO, dichlorodiphenyltrichloroethane (DDT), and malathion at diagnostic doses.

The results of WHO susceptibility and insecticide synergist tests are presented in Fig 2. The mortality rates confirm resistance to 0.03% deltamethrin (29.51–64.79%), 0.75% permethrin (70.72–86.30%), and 4% DDT (46.13–86.47%), across all *Ae. albopictus* populations, with exceptions observed in Lion Rock (90.44% mortality to permethrin; suspected resistance) and North New Territories (97.67% mortality to DDT; suspected resistance). Populations from Hong Kong Island (99.67%) and Lamma Island (99.66%) remain susceptible to 5% malathion, while Lion Rock (91.67%), North New Territories (94.21%), and West New Territories (97.51%) exhibit reduced susceptibility. PBO synergism fully restored susceptibility to deltamethrin in Hong Kong Island (99.32%) and Lamma Island (98.99%) populations, partially restored efficacy in Lion Rock (95.88%), and increased mortality without full restoration in North New Territories (88.2%) and West New Territories (85.78%). For permethrin, PBO achieved compete susceptibility restoration (98.02–100% mortality) in Lamma Island, Lion Rock, and West New Territories populations, with near-complete restoration in Hong Kong Island (97.93%) and North New Territories (97.66%). The mortality rates for all tests with the ATMNJ-95 strain ranged from 98% to 100%. Original data were provided in S4 Table.

Statistical analysis of susceptibility tests revealed that insecticide type was the primary determinant of mortality, with a highly significant main effect (*F*(3,49) = 46.74, *p* < 0.001). In contrast, the random effect of population was not significant (*χ²* [1] = 0.00, *p* = 1.000), accounting for only 2.90% of total variance, indicating minimal geographic variation in resistance patterns. Population density alone showed no significant effect (*F*(1,3) = 4.31, *p* = 0.129), and the insecticide × density interaction approached marginal significance (*F*(3,49) = 2.72, *p* = 0.054). In synergist bioassays, pre-exposure to PBO caused a dramatic 55.4% increase in mortality (*t*(24) = 15.02, *p* < 0.001) for del*t*amethrin. Permethrin showed a smaller but significant PBO effect, with a 18.4% mortality increase (*t*(24) = 9.56, *p* < 0.001).

### *kdr* detection

**Fig 3:**
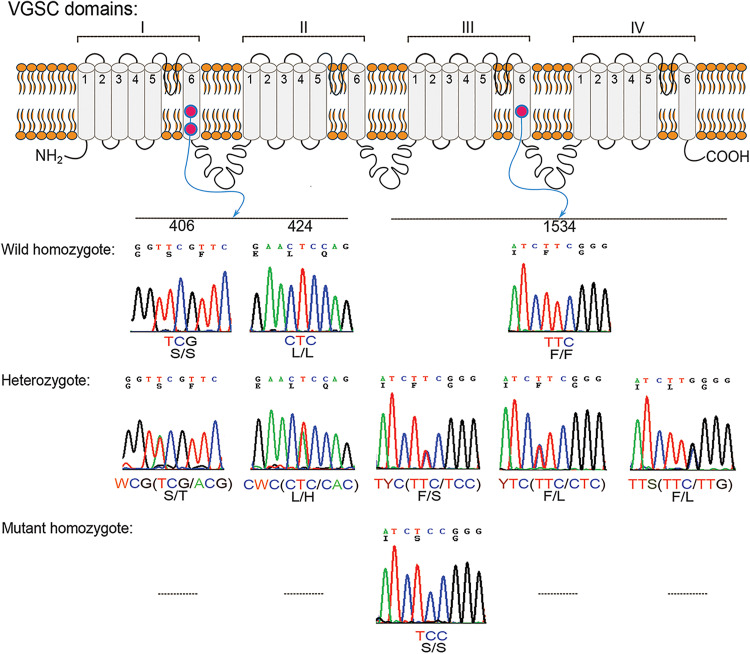
Examples of chromatograms showing non-synonymous mutations in the voltage-gated sodium channel (VGSC) domains of *Ae. albopictus.*

Sequences of VGSC I-IVTm6 from tested samples were deposited in GenBank, with accession numbers PV822561–3277. In VGSC IIITm6, the mutations F1534 (S or L) were identified in both homozygous and heterozygous forms, with a mutation frequency of 29.0% only in Hong Kong Island population (Fig 3 and Table 3). These mutations were significantly associated with pyrethroid resistance (*χ*^*2 *^= 6.48, *p* = 0.01, OR (95% CI) = 3.0 (1.27–7.10)) (Table 4). In VGSC ITm6, we detected the mutations S406T and L424H, which occurred together in heterozygous haplotypes (S406T + L424H) with a mutation frequency of 1.60% (Fig 3 and Table 3) only in Lamma Island Populations. This combination was documented for the first time in mosquitoes. However, these mutations showed no significant association with pyrethroid resistance (*χ*^*2*^^* *^= 2.10, *p* = 0.15, OR (95% CI) = 0.2 (0.01–4.25)) (Table 4). No other reported *kdr* mutations in the *Ae. albopictus* VGSC genes were found in our study (Table 4). Detection of synonymous mutations and intron polymorphisms in *Ae. albopictus* VGSC gene were summarized in S2 File.

**Table 3:** Distribution and frequency of non-synonymous mutations in the voltage-gated sodium channel (VGSC) domains of *Ae. albopictus* populations from Hong Kong.

Population		ITm6			IITm6						IIITm6					IVTm6
		S406T	S406T	L424H	L982W	S989P	S1000Y	A1007G	I1011G	V1016G	T1520I	I1532T	F1534C/S/L			D1763Y
	Genotype												FS	FL	SS	
Hong Kong Island	No.	0	0	0	0	0	0	0	0	0	0	0	22	6	3	0
	Total No.	65	65	65	59	59	59	59	59	59	107	107	107			100
	Mutation Frequency	0%	0%	0%	0%	0%	0%	0%	0%	0%	0%	0%	29%			0%
	Genotype	ST		LH												
Lamma Island	No.	2	0	2	0	0	0	0	0	0	0	0	0			0
	Total No.	126	126	126	54	54	54	54	54	54	80	80	80			73
	Mutation Frequency	1.6%	0%	1.6%	0%	0%	0%	0%	0%	0%	0%	0%	0%			0%

Mutation nomenclature indicates the wild-type amino acid (single-letter code), the codon position, and the mutant amino acid. Frequencies were calculated from a total number of sequenced mosquitoes per domain.

**Table 4:** Assessment of the association between *Ae. albopictus* genotypes and post-pyrethroids bioassays survivorship (phenotype).

Codon site	Genotype	Phenotype					
		No. Live (%)	No. Dead (%)	*χ* ^*2*^	df	*p*	OR (95% CI)
406 + 424	Mutant (S406T + L424H)	0 (0)	2 (1.6)	2.10	1	0.15	0.2 (0.01–4.25)
	Wild	64 (50.8)	60 (47.6)				
1534	Mutant (F1534S/L)	18 (16.8)	13 (12.1)	6.48	1	0.01	3.0 (1.27–7.10)
	Wild	24 (22.4)	52 (48.6)				

The ‘Phenotype’ groups ‘Live’ and ‘Dead’ represent the number and percentage of mosquitoes that survived or died, respectively, after a 24-hour holding period following a 1-hour exposure to pyrethroids. Genotypes were determined via Sanger sequencing. The association was assessed using a chi-square test with degrees of freedom (df) and *p*-values reported. Effect sizes are presented as odds ratios (OR) with 95% confidence intervals (CI).

## Discussion

Our study represents the first research study of the levels and distribution of insecticide resistance in the dengue vector *Ae. albopictus* across Hong Kong, accompanied by an initial examination of the underlying resistance mechanisms of local populations. Specifically, we documented high resistance to pyrethroids and DDT with emerging malathion resistance in Hong Kong *Ae. albopictus* populations, with minimal geographic variation and no discernible trend across the human population density gradient. Emerging resistance threatens the efficacy of spinosad, temephos and pyriproxyfen, whereas *Bti* remains a reliable control agent. It is worth noting that these field-evolved resistance levels could be underestimated by testing on the F2–F3 laboratory-reared progeny due to relaxed insecticide selection pressure and laboratory acclimation effects [75]. Furthermore, we identified two types of non-synonymous mutations, F1534S and F1534L, in VGSC IIITm6, which are contributing to the observed resistance to pyrethroids in Hong Kong Island population. The restoration of susceptibility following PBO treatment provides evidence that metabolic mechanism is instrumental in the development of insecticide resistance in Hong Kong. Our results suggest that the resistance mechanisms observed in Hong Kong’s *Ae. albopictus* populations appear to be multifaceted, involving both *kdr* mutations and metabolic adaptations.

Insecticide resistance in Hong Kong’s mosquito populations exhibits geographical homogeneity, with overall patterns reflected by historical usage trends. The pronounced cross-resistance to pyrethroids and DDT likely stems from shared target-site mutations (e.g., *kdr*) in VGSC gene, exacerbated by prolonged selection pressure from decades of DDT and subsequent pyrethroid deployment [33,41]. Conversely, the relatively low resistance to organophosphates may reflect both a limited historical deployment period and regulatory restrictions imposed due to their broad-spectrum toxicity to non-target aquatic and terrestrial ecosystems [76]. Nevertheless, localized departures are evident. Hong Kong Island and Lamma Island populations demonstrate strong pyrethroid/DDT resistance with retained malathion susceptibility, while Lion Rock is developing resistance to organophosphates (malathion and temephos). The divergent resistance profile observed in New Territories (elevated pyrethroid resistance, reduced DDT resistance) may reflect diminished glutathione S-transferase (GST) activities, as these enzymes are known to metabolize DDT [77]. Additionally, human population density does not appear to affect insecticide resistance. This may reflect selection pressure from agricultural pesticides, which are frequently in the same chemical classes as public health insecticides, meaning rural and countryside areas may show resistance levels comparable to densely populated areas. These findings underscore the need for regionally tailored resistance management strategies informed by continuous resistance monitoring.

In regions and countries adjacent to Hong Kong, comparable resistance mechanism patterns have been documented, indicating a broader regional trend in the evolution and emergence of insecticide resistance among *Aedes* populations, albeit with certain distinctions. Resistance to pyrethroids has been observed in southern China in the provinces of Guangdong, Guangxi, Hainan, Yunnan, Zhejiang, and Fujian [69,78]. Investigations conducted in Guangzhou, situated 158 kilometers from Hong Kong, have demonstrated significant correlations between mutations F1534S and F1534 L of the VGSC gene and pyrethroid resistance [34,79]. Similar findings have been reported in other provinces contiguous to Guangdong, with the mutation at codons F1534 and I1532 being associated with resistance [69,80–82]. In Vietnam, the V1016G allele exerts a more pronounced influence on insecticide resistance than F1534C or F1534S [68]. In Laos, although V1016G and F1534C have been detected in *Ae. aegypti* populations, they do not exhibit a significant association with survivorship to DDT or pyrethroids. Instead, elevated activities of cytochrome P450 monooxygenases were significantly correlated with insecticide resistance [83]. Similar phenomena have been reported from other Southeast Asian countries, such as Cambodia and Malaysia [65,84]. Nevertheless, *kdr* mutations, especially at codons 1534 and 1016, have been documented in *Aedes* populations worldwide [43,44]. When combined with cytochrome P450 overexpression [30,85], these mechanisms synergistically drive insecticide resistance on a global scale, significantly compromising the efficacy of vector control initiatives.

Current evidence suggests that pyrethroid resistance in *Ae. albopictus* cannot be fully explained by *kdr* mutations alone; instead, metabolic detoxification mechanisms are important contributors. In this study, *kdr* mutations were detected only in Hong Kong Island. Given the PCR assay’s poor sensitivity and high failure rates, we restricted *kdr* genotyping to two populations to avoid protracted, low-yield analyses. Future work should prioritize next-generation sequencing (NGS) of the VGSC gene to achieve unbiased, genome-wide resistance allele detection. Crucially, the restoration of pyrethroid susceptibility through pre-exposure to PBO strongly implicates cytochrome P450-mediated pathways as critical resistance determinants. Further corroborating this, recent studies have identified specific P450 enzymes directly linked to resistance: CYP6P12 overexpression has been mechanistically validated as a driver of pyrethroid resistance in both *Ae. albopictus* and *Ae. aegypti* [83,86], while CYP9J32 contributes to resistance in Vietnam *Ae. aegypti* populations [87]. CYP6BB2 has been functionally associated with both DDT and pyrethroid resistance in *Ae. aegypti* in Laos [83]. Furthermore, genomic analyses reveal extensive molecular adaptations, including 41 gene amplifications (predominantly P450-related) and 55 nonsynonymous variants in *Ae. aegypti* [88]. Nevertheless, key knowledge gaps persist regarding epigenetic regulation (e.g., *N*^6^-methyladenosine (m^6^A) RNA) [89] and cis-regulatory mutations (e.g., *Culex* Repetitive Element 1 (*CuRE1*) [90] in P450 genes. The complexities of metabolic mechanisms highlight the necessity for integrated multi-omics approaches to decode the polygenic architecture of insecticide resistance, thereby enabling precision vector control strategies [91].

Evidence demonstrated that *Aedes* vectors have been under the process of developing resistance to organophosphates worldwide, despite underlying mechanisms remain insufficiently studied. A striking example is the rapid evolution of malathion resistance in Brazil *Ae. aegypti* populations within a mere two-year period (2016–2018) [92]. Organophosphate resistance in this species has been documented across diverse regions, including other South American countries [93,94] and Indonesia [95]. Similarly, *Ae. albopictus* populations exhibit resistance in Laos [96] and the United States [35,97] and China [81,98], and both species show resistance in Thailand [99]. Their resistance mechanisms might involve metabolic detoxification via elevated carboxylesterase (e.g., *CCEae3a*, *CCEae6a*) and cytochrome P450 gene (e.g., *Cyp6z18*, *Cyp6d4*) in *Ae. albopictus* [100–102]. Target-site mutations such as G119S, F290V, and F331W substitutions in acetylcholinesterase-1 (*ace-1*) have been reported but only in *Anopheles* and *Culex* mosquitoes [103–106]. Additional mechanisms, such as cuticular thickening further contributes to resistance [102]. Notably, cross-resistance between organophosphates and pyrethroids is likely to be mediated by shared detoxification pathways, particularly cytochrome P450 upregulation [107,108].

The emergence of resistance to different larvicides in Hong Kong *Aedes* populations is consistent with trends observed in other regions worldwide. Recent studies reveal species- and region-specific patterns: *Ae. albopictus* in China exhibits moderate to high resistance to pyriproxyfen [34], while *Ae. aegypti* in Laos and Cambodia shows tolerance to spinosad [109,110]. Temephos resistance, widespread in *Ae. aegypti* across Latin America and South Asia [32], has also been detected in *Ae. albopictus* populations in China [34] and Laos [96]. Additionally, evidence suggests the possibility of cross-resistance between temephos and spinosad in *Ae. aegypti* populations in Brazil [111], which may explain the emerging resistance to both insecticides observed in the Lion Rock population. In contrast, *Bti* retains high efficacy globally, with no confirmed resistance to date. The mechanisms driving larvicide resistance remain poorly characterized, though cross-resistance patterns are increasingly evident. For instance, *Ae. albopictus* populations in Fujian and Guangdong provinces display cross-resistance between pyrethroids and pyriproxyfen, linked to shared metabolic pathways [34,80]. Another study implicates cytochrome P450 enzymes in conferring resistance to pyrethroids, organophosphates, and pyriproxyfen [112], suggesting that overexpression of these detoxification systems may drive broad-spectrum resistance to both larvicides and adulticides. This cross-resistance phenomenon is likely to develop within the Hong Kong Island population. Furthermore, the broad confidence interval observed for pyriproxyfen indicates heterogeneity within the tested population, likely due to the stage-dependent mode of action of pyriproxyfen—a juvenile hormone analog that tends to be less effective in late-instar larvae than in early instars [113]. All these findings emphasize the urgency of elucidating resistance mechanisms to optimize integrated vector management, mitigate the risk of multi-insecticide resistance and guide effective larvicide application.

## Conclusion and recommendation

The findings of this study reveal a concerning severity of insecticide resistance in *Ae. albopictus* populations across Hong Kong, particularly to pyrethroids. The observed resistance, likely driven by multifaceted mechanisms including metabolic detoxification and *kdr* mutations, compromises the efficacy of current dengue prevention strategies. The observed resistance patterns in Hong Kong aligned with reported in adjacent regions and countries [65,68,69,78,82,83,86], suggesting a contiguous hotspot in southern China and Southeast Asia. This regional convergence of resistance mechanisms complicates vector management and elevates the difficulty to control dengue and other arboviral disease outbreaks. This challenge is exacerbated by increasing urbanization and climate change, which continue to expand the habitats for *Ae. albopictus* populations [114,115]. To mitigate the resistance, sustained resistance surveillance is essential. In parallel, we can implement rotational schemes that alternate pyrethroids with non-pyrethroid insecticides, or combine pyrethroids with synergists like PBO, or emphasize biological control through the application of biolarvicides (e.g., *Bti*) or *Wolbachia* [116] to suppress *Ae. albopictus* populations. New insecticides which have been recently prequalified by WHO can be good alternatives to the currently used insecticides for Public Health vector control, such as clothianidine (neonicotinoide family) and broflanilide, a new molecule from the family of meta-diamide [117]. These insecticides are not yet registered in Hong Kong [39]. Future research should involve efficacy testing on local mosquito populations to support registration with Hong Kong authorities.

## Supporting information

S1 Table Specific locations for ovitraps. (XLSX)

S2 Table List of primer sets used for the PCR and sequencing of the VGSC gene. (XLSX)

S3 Table Data of larval bioassays. (XLSX)

S4 Table Data of adult bioassays. (XLSX)

S1 File Dose-response curves of larval bioassays. (DOCX)

S2 File Synonymous mutations and intron polymorphisms of VGSC I-IVTm6. (DOCX)
