# Supplementary material for: Insecticide resistance levels, spatial distribution, and kdr mutations in the dengue vector Aedes albopictus of Hong Kong
Source: PLoS Negl Trop Dis. 2025 Dec 22;19(12):e0013792. doi: 10.1371/journal.pntd.0013792 (PMC12721537; doi:10.1371/journal.pntd.0013792)
Supplement: S1 File — (DOCX) [file pntd.0013792.s005.docx]

**Supplementary Materials for “Insecticide resistance levels, spatial distribution, and *kdr* mutations in the dengue vector *Aedes albopictus* of Hong Kong”**

Shaolin Han^1,2¶^, Elliott F. Miot^1,3¶^, Yunshi Liao^1,2¶^, Munsif Ali Khan^1,2^, Mathilde Rivot^1^, Lilia Tsz-Wing Tang^1^, Jehan Zeb^1^, Ka Mei Szeto^1^, Long Ching^1^, Tsz Him Li^1^, Xintong Huang^2^, Brinna E. L. Barlow^1,2^, Sebastien Marcombe^1,4*^, Tommy Tsan-Yuk Lam^1,2,5,6,7*^

^1^Centre for Immunology & Infection, Hong Kong SAR, China

^2^State Key Laboratory of Emerging Infectious Diseases, School of Public Health, The University of Hong Kong, Hong Kong SAR, China

^3^MIVEGEC, Université de Montpellier, IRD, CNRS, 34394 Montpellier, France

^4^Vector Control Consulting—South East Asia Sole Co., Ltd., Vientiane, Laos

^5^Laboratory of Data Discovery for Health, Hong Kong SAR, China

^6^HKU-Pasteur Research Pole, Hong Kong SAR, China

^7^The Hong Kong Jockey Club Global Health Institute, The University of Hong Kong, Hong Kong SAR, China

^*^[sebastienmarcombe@gmail.com](mailto:sebastienmarcombe@gmail.com); [ttylam@hku.hk](mailto:ttylam@hku.hk)

^¶^These authors contributed equally to this work.

**Supplementary Materials**

*Dose-response Curves*

All dose-response relationships are presented in Fig 1-4.


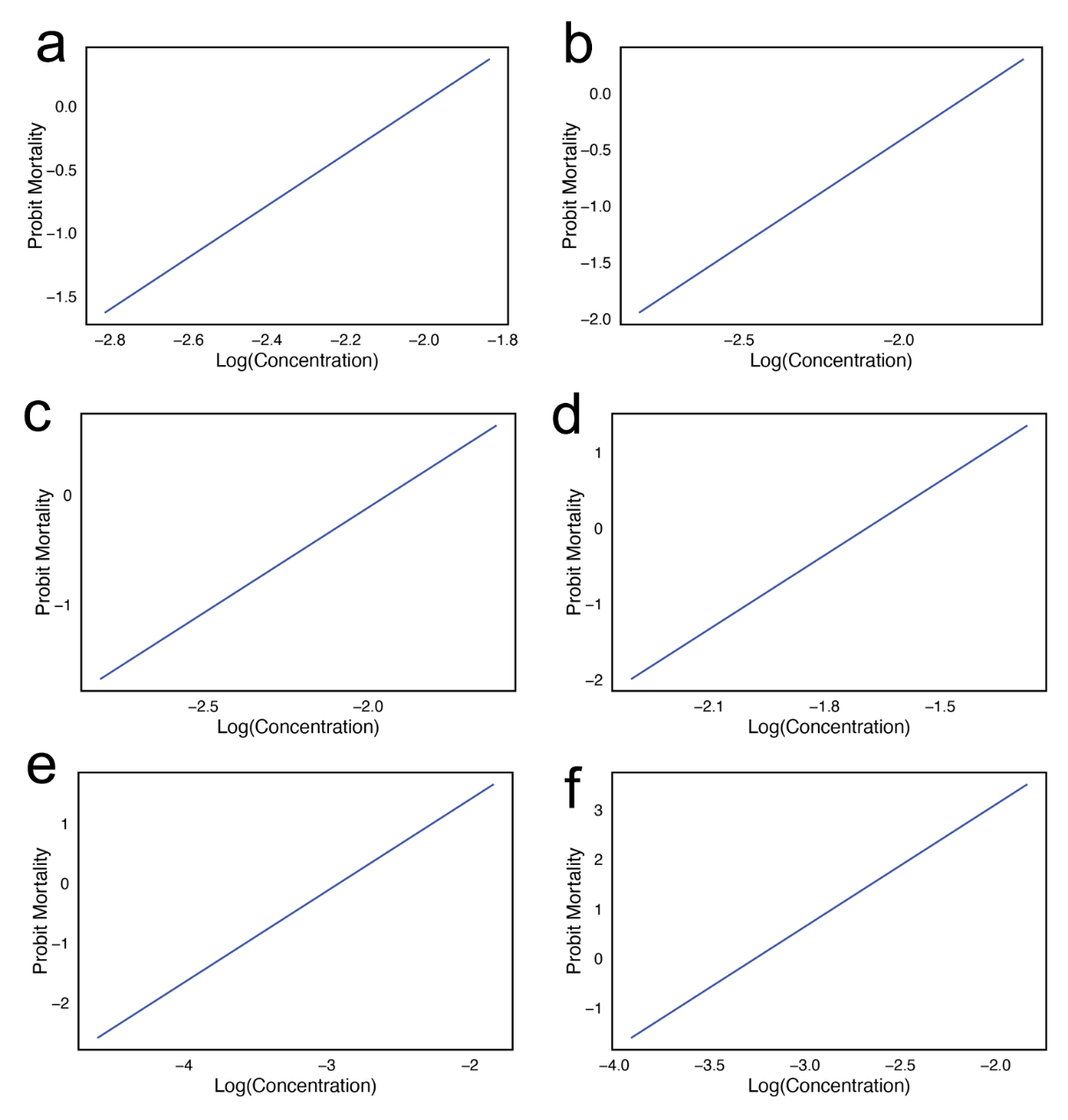


Fig 1. Dose-response of a) ATM-NJ95 strain; b) Hong Kong Island population; c) Lamma Island population; d) Lion Rock population; e) North New Territories population; f) West New Territories population to *Bti*.


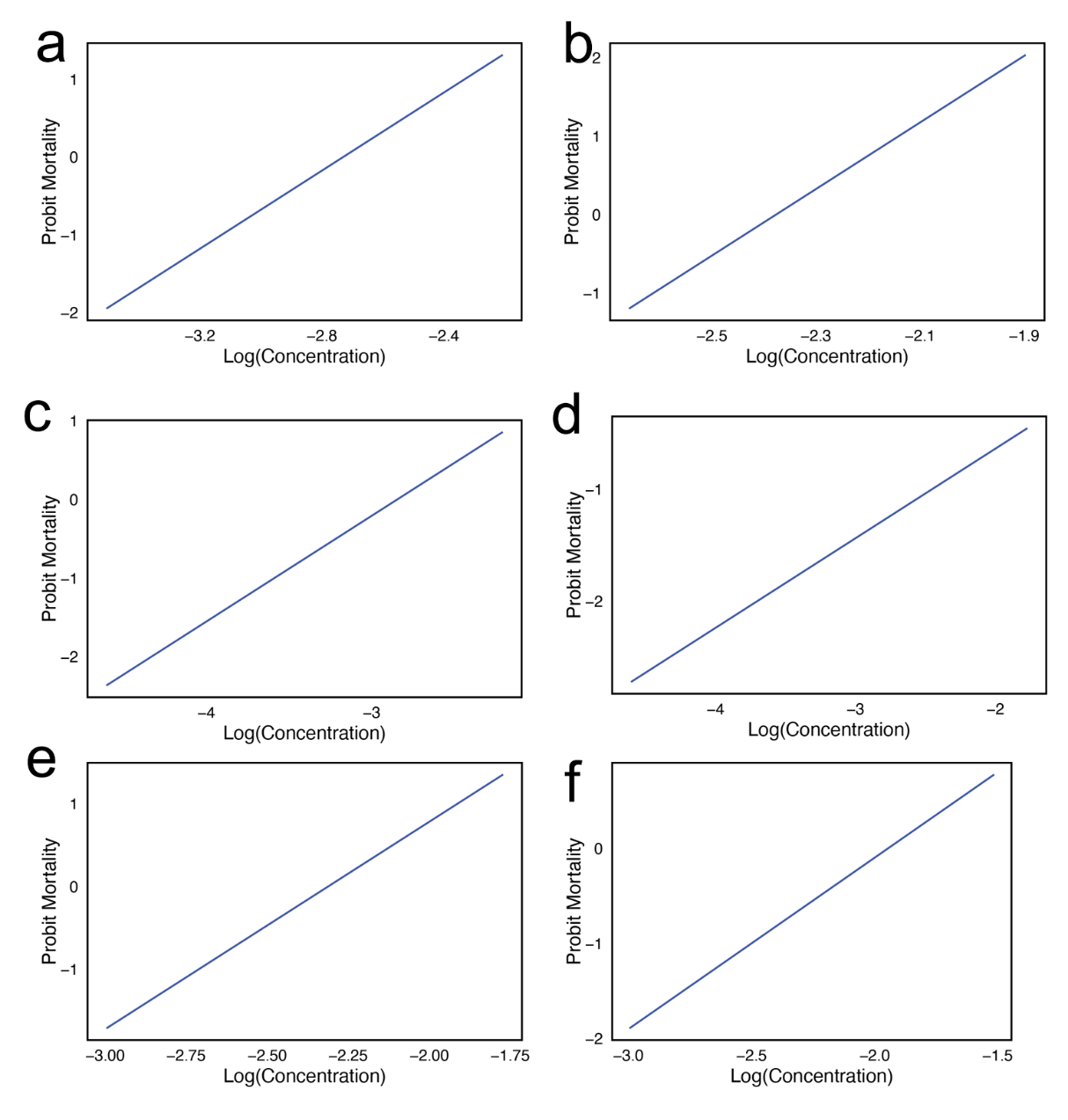


Fig 2. Dose-response of a) ATM-NJ95 strain; b) Hong Kong Island population; c) Lamma Island population; d) Lion Rock population; e) North New Territories population; f) West New Territories population to spinosad.


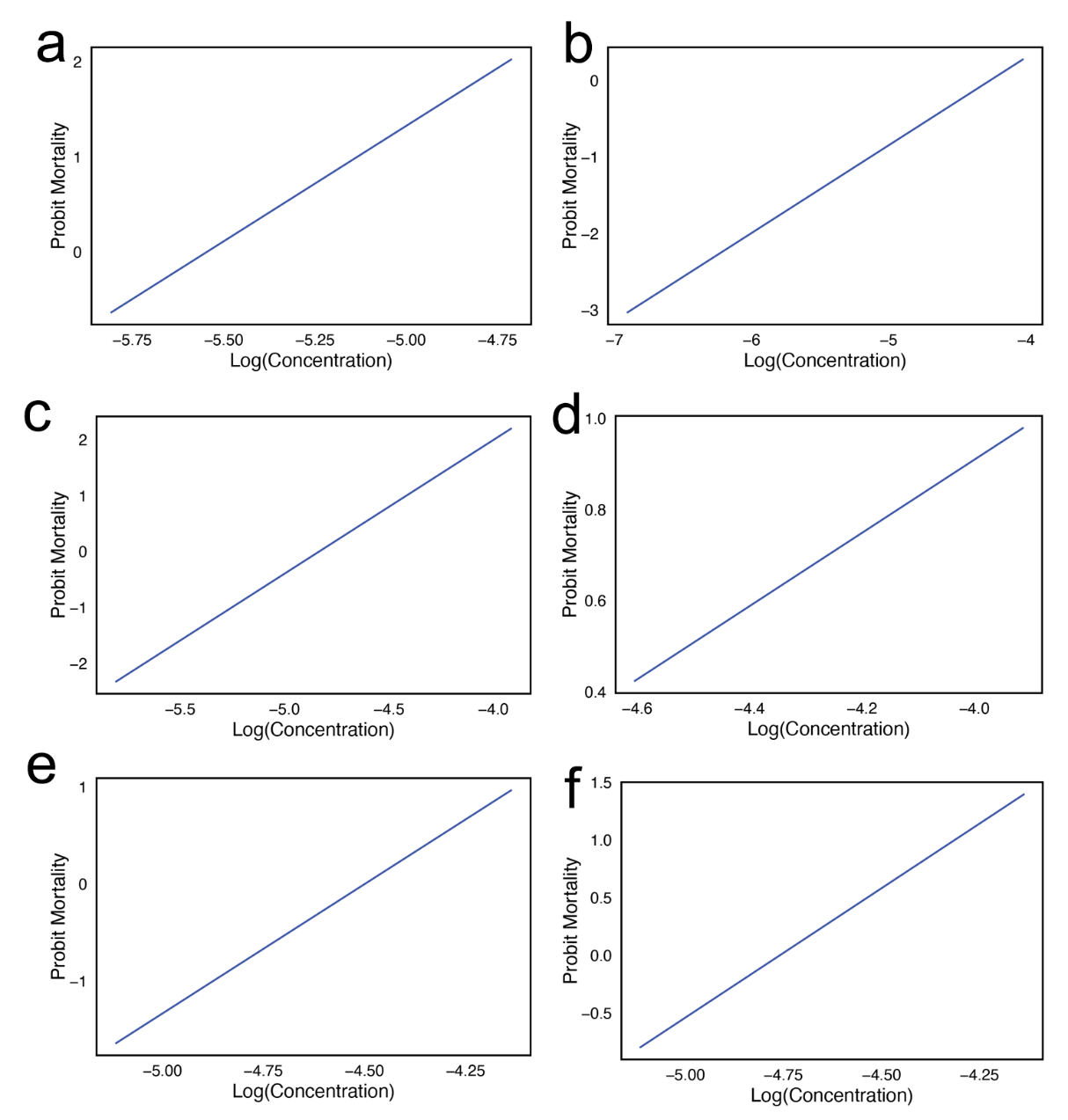

Fig 3. Dose-response of a) ATM-NJ95 strain; b) Hong Kong Island population; c) Lamma Island population; d) Lion Rock population; e) North New Territories population; f) West New Territories population to temephos.


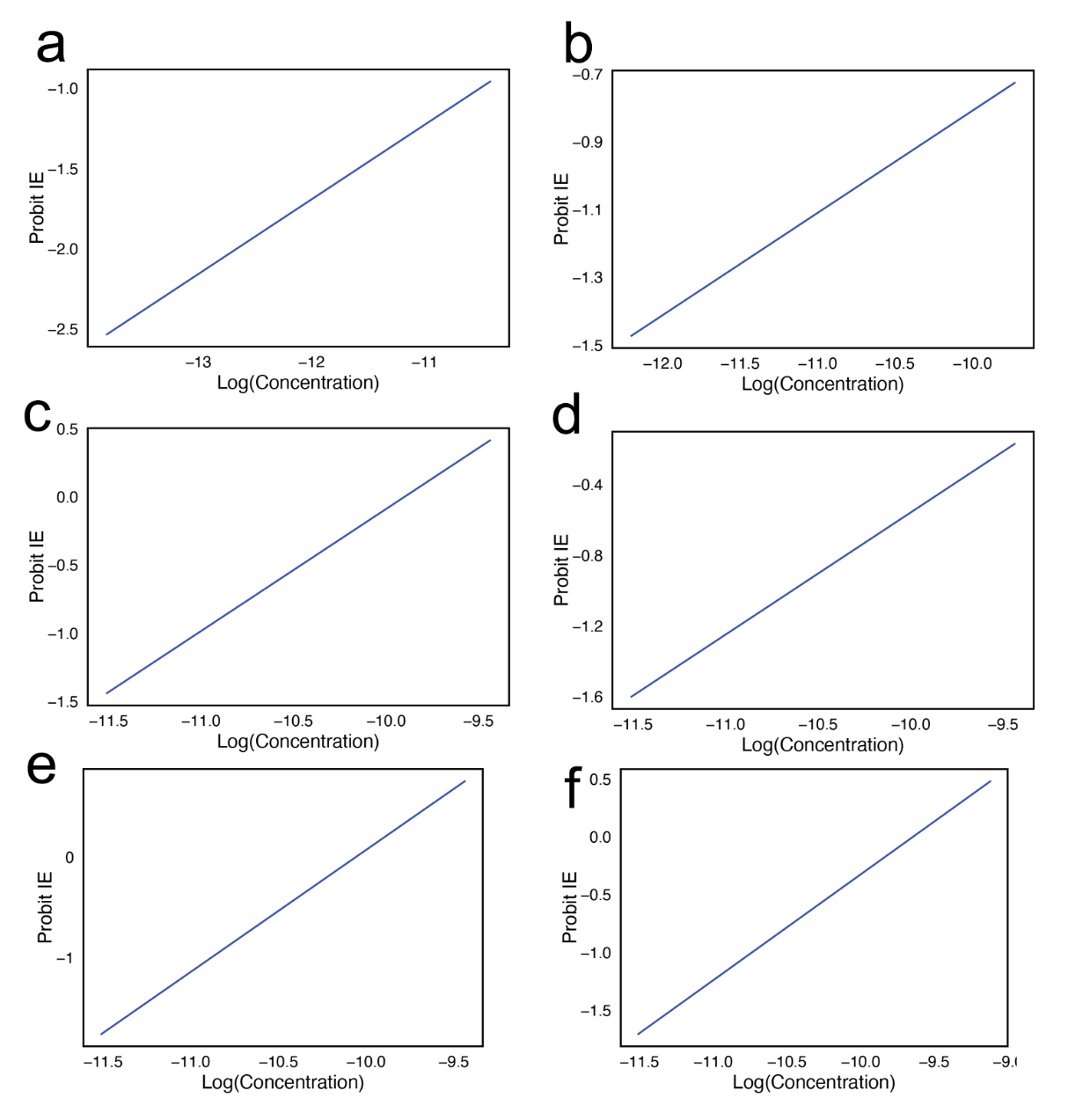


Fig 4. Dose-response of a) ATM-NJ95 strain; b) Hong Kong Island population; c) Lamma Island population; d) Lion Rock population; e) North New Territories population; f) West New Territories population to pyriproxyfen.
