# Supplementary material for: Insecticide resistance levels, spatial distribution, and kdr mutations in the dengue vector Aedes albopictus of Hong Kong
Source: PLoS Negl Trop Dis. 2025 Dec 22;19(12):e0013792. doi: 10.1371/journal.pntd.0013792 (PMC12721537; doi:10.1371/journal.pntd.0013792)
Supplement: S2 File — (DOCX) [file pntd.0013792.s006.docx]

**Supplementary Materials for “Insecticide resistance levels, spatial distribution, and *kdr* mutations in the dengue vector *Aedes albopictus* of Hong Kong”**

Shaolin Han^1,2¶^, Elliott F. Miot^1,3¶^, Yunshi Liao^1,2¶^, Munsif Ali Khan^1,2^, Mathilde Rivot^1^, Lilia Tsz-Wing Tang^1^, Jehan Zeb^1^, Ka Mei Szeto^1^, Long Ching^1^, Tsz Him Li^1^, Xintong Huang^2^, Brinna E. L. Barlow^1,2^, Sebastien Marcombe^1,4*^, Tommy Tsan-Yuk Lam^1,2,5,6,7*^

^1^Centre for Immunology & Infection, Hong Kong SAR, China

^2^State Key Laboratory of Emerging Infectious Diseases, School of Public Health, The University of Hong Kong, Hong Kong SAR, China

^3^MIVEGEC, Université de Montpellier, IRD, CNRS, 34394 Montpellier, France

^4^Vector Control Consulting—South East Asia Sole Co., Ltd., Vientiane, Laos

^5^Laboratory of Data Discovery for Health, Hong Kong SAR, China

^6^HKU-Pasteur Research Pole, Hong Kong SAR, China

^7^The Hong Kong Jockey Club Global Health Institute, The University of Hong Kong, Hong Kong SAR, China

^*^[sebastienmarcombe@gmail.com](mailto:sebastienmarcombe@gmail.com); [ttylam@hku.hk](mailto:ttylam@hku.hk)

^¶^These authors contributed equally to this work.

**Supplementary Materials**

*Synonymous mutations*

The silent mutations identified across all domains of *Ae. albopictus* VGSC amplicons were VGSC-ITm6 I416I (ATY: ATT/C) and E435E (GAR: GAG/A); VGSC-IITm6 V981V (GTR: GTA/G), C983C, C993C, C1001C (TGY: TGT/C), and F1004F (TTY: TTC/T); VGSC-IIITm6 C1466C (TGY: TGC/T), E1474E (GAR: GAG/A), S1476S (TCR: TCG/A), P1477P, P1516P (CCR: CCG/A), G1484G (GGR: GGG/A), Y1487Y, Y1523Y (TAY: TAC/T), A1506A (GCY: GCC/T), R1510R (CGR: CGG/A), V1512V (GTR: GTG/A), I1517I (ATW: ATT/A), F1528F (TTY: TTC/T), L1540L (CTY: CTC/T), N1541N (AAY: AAC/T), I1548I, I1781I (ATY: ATC/T), and G1559G (GGY: GGT/C); VGSC-IVTm6 L1726L, L1736L, L1745L, L1806L (YTG: C/TTG)/(CTR: CTG/A), F1727F, F1750F, F1773F (TTY: TTC/T)/(TTY: TTT/C), A1735A, A1790A (GCM: GCC/A)/(GCY: GCC/T), I1749I, I1799I, I1823I, I1825I, I1834I (ATY: ATC/T)/(ATW: ATA/T), G1754G, G1766G, G1791G, G1798G, G1819G, G1824G (GGS: GGG/C)/(GGR: GGG/A)/(GGW: GGA/T)/(GGY: GGC/T), S1765S, S1779S, S1789S, S1835S (AGY: AGC/T)/(TCK: TCG/T), N1772N (AAY: AAT/C), K1774K, K1812K (AAR: AAG/A), Q1778Q (CAR: CAG/A), T1788T, T1826T (ACR: ACG/A), V1795V (GTR: GTG/A), E1802E, E1804E (GAR: GAG/A)/(GAR: GAA/G), C1805C, C1818C (TGY: TGC/T), P1807P, P1808P (CCY: CCT/C)/(CCK: CCG/T)/(CCR: CCG/A), D1811D (GAY: GAC/T), and Y1814Y, Y1827Y (TAY: TAC/T). Most synonymous mutations observed across the VGSC domains appeared heterozygous, while few were found as homozygous silent mutations.

Domain-wise analysis showed that synonymous mutations were more frequently observed in VGSC-IVTm6, followed by VGSC-IIITm6, VGSC-IITm6, and VGSC-ITm6, respectively. Transition-type base substitution was more common than transversion among the synonymous mutations detected in the VGSC amplicons.

*Intron polymorphisms*

Intron polymorphism analysis of the *Ae. albopictus* VGSC dataset revealed three intervening introns with variable length in each respective VGSC domain: intron 20 flanked by partial exons 20 and 21 in VGSC-IITm6; intron 28 flanked by partial exons 28 and 29; and intron 30 flanked by partial exons 30 and 31. Four polymorphic forms of intron 20 were observed with variable length, viz. L+ (90 bp, 56.63%), L++ (91 bp, 7.96%), S- (82 bp, 32.74%), S-- (71 bp, 2.65%) compared to the *Ae. albopictus* intron 20 reference sequence (89 bp) (Gene ID: 109421922) (see Fig 1). However, previously reported longer intron 20 (group B) in *Ae. albopictus* from Cambodia was absent in our sequenced dataset [1]


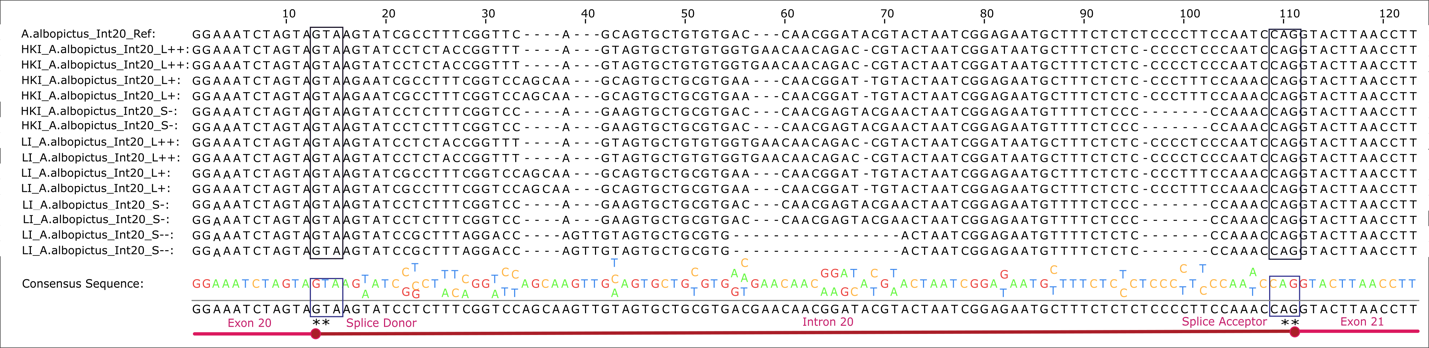
Fig 1. Polymorphism in VGSC-IITm6 Intron 20 (flanked by partial exons 20 and 21) based on its length (bp) and sequence variation. The MSA file illustrates lengthwise variations among the sequenced haplotypes. Asterisks denote splice donor and acceptor sites.

For intron 28, we found three polymorphs among the VGSC-IIITm6 amplicons that were either identical (E: 83 bp, 92.51%) to/or shorter (S-: 70 bp, 2.67%; S--: 68 bp, 4.81%) than the *Ae. albopictus* intron 28 reference sequence (83 bp) (Gene ID: 109421922) (Fig 2).


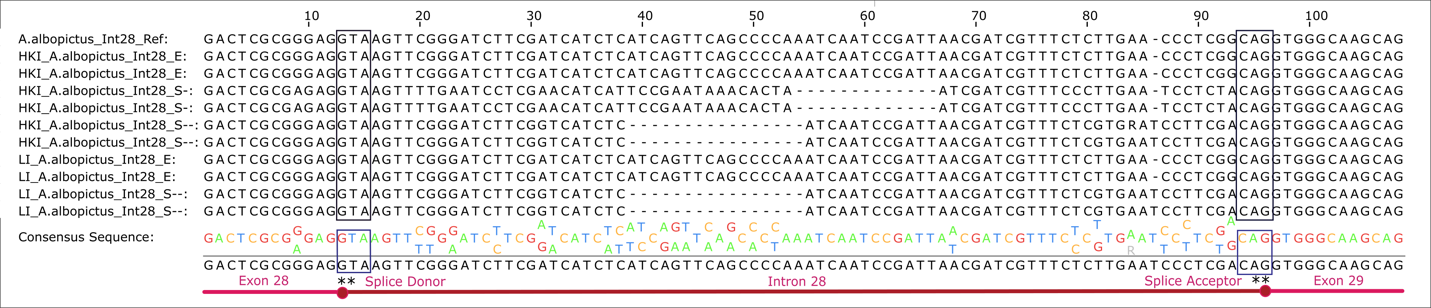
Fig 2. Polymorphism in VGSC-IIITm6 Intron 28 (flanked by partial exons 28 and 29) based on its length (bp) and sequence variation. The MSA file illustrates lengthwise variations among the sequenced haplotypes. Asterisks denote splice donor and acceptor sites.

Similarly, three polymorphic variants for intron 30 were noticed in the VGSC-IVTm6 amplicons. These polymers were either alike (E: 64 bp, 90.69%) to/or longer (L+: 66 bp, 4.56%)/shorter (S-: 63 bp, 4.56%) in length than the *Ae. albopictus* intron 30 reference sequence (64 bp) (Gene ID: 109421922) (Fig 3).


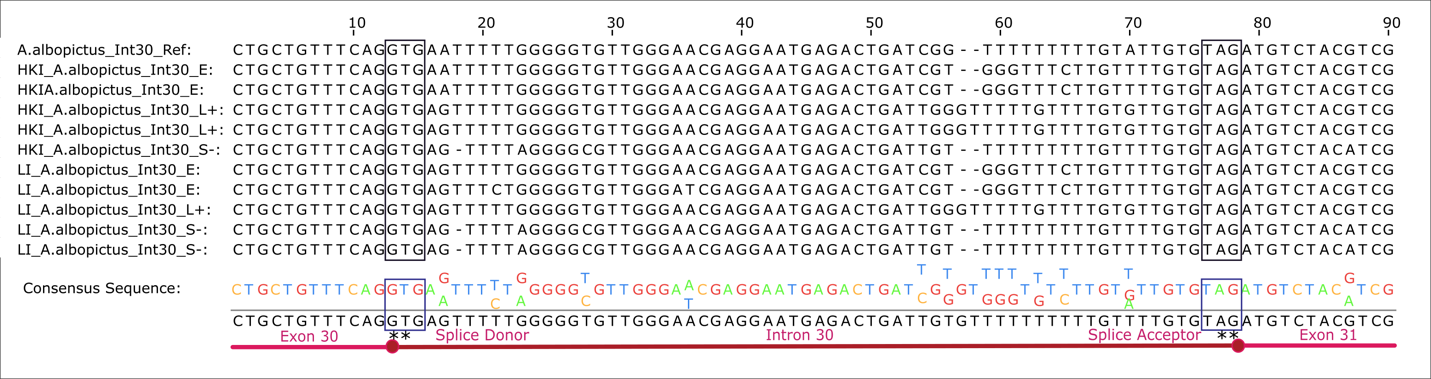
Fig 3. Polymorphism in VGSC-IVTm6 Intron 30 (flanked by partial exons 30 and 31) based on its length (bp) and sequence variation. The MSA file illustrates lengthwise variations among the sequenced haplotypes. Asterisks denote splice donor and acceptor sites.

Mutation detection analysis showed the presence of mutations across the characterized introns, 20, 28, and 30. However, the splice donor and acceptor sites were found intact, and no frameshift mutation in the subsequent exons was observed.

**References**

1. Marcombe S, Doeurk B, Thammavong P, Veseli T, Heafield C, Mills M-A, et al. Metabolic resistance and not voltage-gated sodium channel gene mutation is associated with pyrethroid resistance of *Aedes albopictus* (Skuse, 1894) from Cambodia. Insects. 2024;15(5):358. PubMed PMID: doi:10.3390/insects15050358.
